# Clinicopathological characteristics and survival in lung signet ring cell carcinoma: A population-based study

**DOI:** 10.17305/bjbms.2020.5454

**Published:** 2021-12

**Authors:** Yunting Cai, Yan Xie, Yanli Xiong, Wei Guan, Yu Pu, Dong Wang, Mingfang Xu, Shenglan Meng

**Affiliations:** 1 Department of Thoracic Surgery, Daping Hospital, Army Medical University, Chongqing, China; 2 Cancer Center of Daping Hospital, Army Medical University, Chongqing, China

**Keywords:** Lung signet ring cell carcinoma (LSRCC), SEER, clinical characteristics, prognosis

## Abstract

Lung signet-ring cell carcinoma (LSRCC) is a very rare type of lung cancer, the clinical characteristics, and prognosis of which remain to be clarified. In order to explore the clinicopathological and survival-related factors associated with LSRCC, we performed a large population-based cohort analysis of data included in the Surveillance, Epidemiology, and End Results (SEER) registry from 2001 to 2015. A total of 752 LSRCC and 7518 lung mucinous adenocarcinoma (LMAC) patients were incorporated into our analysis, with respective mean ages of 63.8 and 67.5 years at the time of diagnosis. LSRCC patients were significantly more likely than LMAC patients to have distant-stage disease (72.1% vs. 45.8%, p < 0.0001), tumors of a high pathological grade (40.6% vs. 10.8%, p < 0.0001), have undergone chemotherapy (62.1% vs. 39.9%, p<0.0001), be male (52.7% vs. 48.5%, p = 0.03), and be < 40 years old (3.3% vs. 1.3%, p = 0.022), whereas they were less likely to have undergone surgical treatment (52.4% vs. 77.0%, p < 0.0001). LSRCC and LMAC patients exhibited median overall survival (OS) duration of 8 and 18 months (p < 0.0001), respectively, although these differences were not significant after adjusting for confounding variables. Independent factors associated with a favorable patient prognosis included a primary site in the middle or lower lung lobe, underwent surgery, and underwent chemotherapy. However, age ≥80 years, higher grade, distant summary stage disease, and T4 stage disease were linked to poor prognosis. Patient age, tumor grade, primary tumor site, summary stage, T stage, surgery, and chemotherapy were all significantly associated with LSRCC patient prognosis.

## INTRODUCTION

Signet-ring cell carcinoma (SRCC) is a form of highly aggressive, diffusely invasive cancer that most often originates from the gastrointestinal tract, but that can also develop in breast [1,2], prostate [3,4], and bladder [5] tissues in rare cases. While lung SRCC (LSRCC) primary tumors are extremely rare, they do occur and were first proposed as a unique disease entity with specific clinicopathological characteristics and a poor prognosis by Kish et al. in 1989 [6]. One study of 24,171 SRCC patients found that just 3.1% of these cases originated in the lungs [7]. Multivariate analyses revealed LSRCC to have comparable cause-specific survival to that of gastric SRCC. However, clinical knowledge pertaining to LSRCC is primarily restricted to individual case reports and limited case series, and there have not been any large-scale studies of the clinicopathological characteristics of this disease type or corresponding patient prognosis. Further study of LSRCC is thus warranted to guide appropriate diagnostic and therapeutic efforts.

The Surveillance, Epidemiology, and End Results (SEER) database, developed by the National Institutes of Health, represents the largest and most comprehensive North American cancer database [8], supporting in-depth analyses of rare disease types [9]. As such, we herein leveraged the SEER database to conduct a retrospective analysis of LSRCC cases in an effort to summarize the clinical findings associated with this disease type and to identify factors associated with patient prognosis.

## MATERIALS AND METHODS

### Ethics

The study was conducted in accordance with the Declaration of Helsinki (as revised in 2013). No patient consent was required as these data are public and the authors have signed the SEER research data usage agreement.

### Population

The SEER database (http://seer.cancer.gov/) was queried to identify all patients diagnosed from 2001 to 2015 with mucinous adenocarcinoma (IDC, 8480/3), mucin-producing adenocarcinoma (IDC, 8481/3), and SRCC (IDC, 8490/3) of lung origin as per the ICD-0–3/WHO 2008 criteria. Case identification was conducted with the SEER*Stat 8.3.6.1 software platform, leading to the respective identification of 939 LSRCC and 8613 LMAC patients. Patients were excluded from these analyses if: (1) They had not undergone pathological confirmation of their diagnosis, (2) had been diagnosed after death, (3) had uncertain overall survival (OS) outcomes, (4) were of uncertain ethnicity or marital status, (5) exhibited disease of uncertain summary stage, or (6) had a history of other tumor types. Using these criteria, a total of 752 LSRCC and 7518 LMAC patients were included in subsequent analyses.

### Statistical analysis

Categorical data are presented as numbers with corresponding percentages and were compared using Chi-squared tests. Continuous data are medians or means. OS was compared between patient cohorts based on the time from diagnosis to all-cause death using Kaplan–Meier curves and log-rank (Mantel-Cox) tests. Variables incorporated into models of patient prognosis included age at diagnosis, grade, tumor primary site, summary stage, T stage, N stage, M stage, primary site surgery, radiation, and chemotherapy. Univariate and multivariate Cox proportional hazard models were used to assess LSRCC patient survival-related variables. A two-sided *p* < 0.05 was the significance threshold in this analysis, and SPSS v23 (IBM, NY, USA) was employed for all statistical testing.

## RESULTS

### Patient characteristics

An initial search of the SEER database led to the identification of 939 and 8613 patients diagnosed with LSRCC and LMAC, respectively, from 2001 to 2015. Of these patients, 752 and 7518, respectively, were included in our final analyses, with respective mean ages at diagnosis of 63.8 and 67.5 years. Clinical and demographic information associated with these two patient cohorts is compiled in Table 1.

**TABLE 1:**
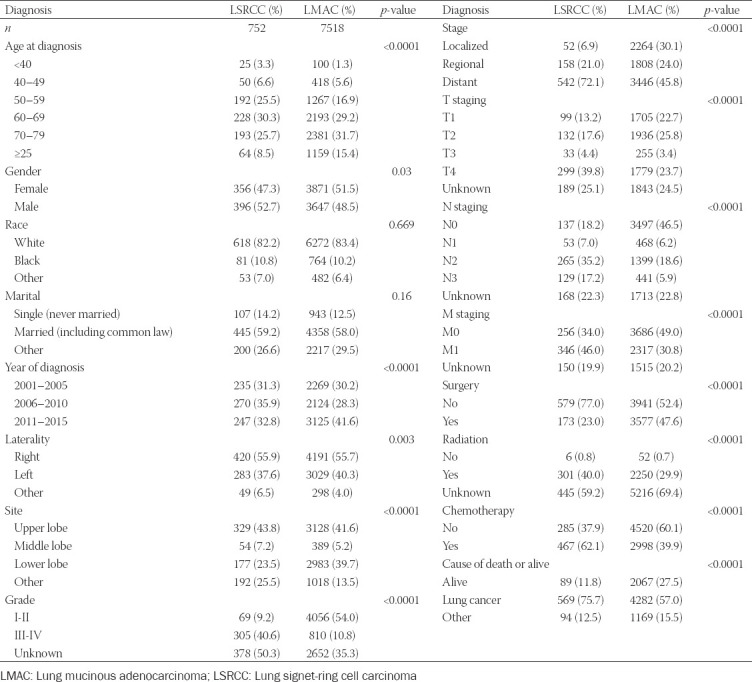
Demographic and clinical characteristics of patients with LMAC and LSRCC between 2001 and 2015 in SEER database

### Survival analysis

We observed significant differences between LSRCC and LMAC patient cohorts with respect to age at diagnosis, gender, lung lobe laterality, primary tumor site, pathological grade, summary stage, T staging, N staging, M staging, primary site surgery status, chemotherapy, and OS. However, marital status and ethnicity did not differ significantly between these two patient groups.

**FIGURE 1:**
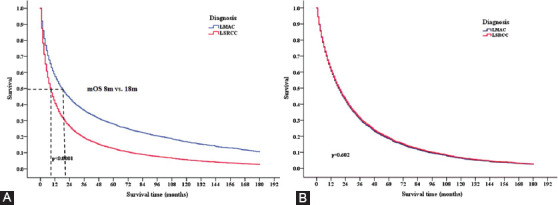
Kaplan–Meier survival analyses of LMAC and LSRCC patients. (A) Kaplan–Meier curves indicated that LSRCC patients had a poorer prognosis than did LMAC patients (8 vs. 18 months; HR 1.62, 95% CI 1.47–1.79, p < 0.0001). (B) Kaplan–Meier curves indicated that the mOS of LSRCC patients did not differ significantly from that of LMAC patients after adjusting for age at diagnosis, site, grade, stage, T staging, N staging, M staging, primary site surgery, radiation, and chemotherapy. LMAC: Lung mucinous adenocarcinoma; LSRCC: Lung signet-ring cell carcinoma; HR: Hazard ratio; CI: Confidence interval; mOS: Median overall survival.

We next compared LSRCC and LMAC patient survival outcomes (Figure 1A). LSRCC patients had a median OS of 8 months (range: 1–191 months), whereas for LMAC patients, the median OS was 18 months (range: 1–185 months) (HR 1.62, 95% CI 1.47–1.79, *p* < 0.0001). However, no significant differences in patient OS were observed between these two patient groups after adjusting for age at diagnosis, site, grade, stage, T staging, N staging, M staging, primary site surgery, radiation, and chemotherapy (Figure 1B).

**FIGURE 2:**
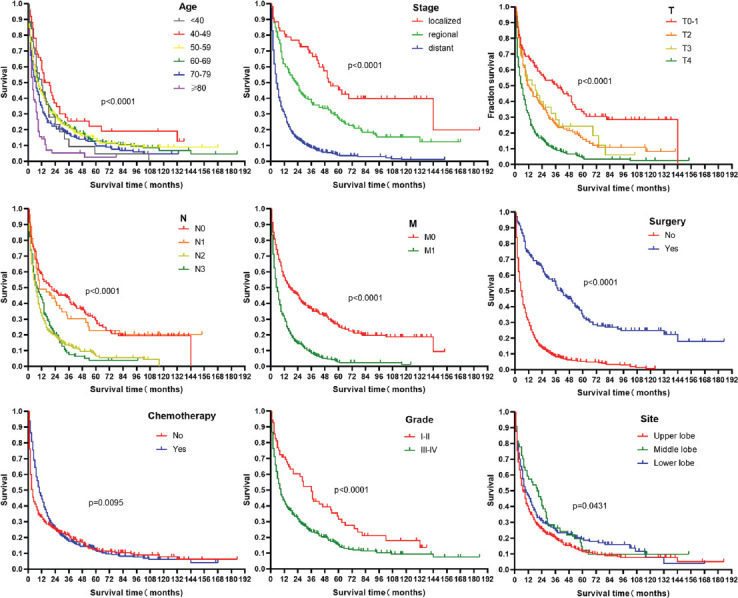
Kaplan–Meier survival curves of lung signet-ring cell carcinoma patients according to age at diagnosis, summary stage, T staging, N staging, M staging, primary site surgery, chemotherapy, and primary tumor site.

We then compared LSRCC patient survival outcomes as a function of age at diagnosis, summary stage, T staging, N staging, M staging, primary site surgery, chemotherapy, and primary tumor site (Figure 2). Kaplan–Meier curves revealed that patients ≥80 years old at the time of diagnosis exhibited significantly worse survival outcomes relative to other patients. The respective median OS durations of patients with the localized, regional, and distant disease were 53 months, 22 months, and 5 months (*p* < 0.0001). Patients with T1, T2, T3, and T4 stage disease exhibited median OS durations of 37 months, 12 months, 18 months, and 6 months, respectively (*p* < 0.0001), while the median OS of individuals with N0, N1, N2, and N3 staging was 21 months, 10 months, 7 months, and 8 months, respectively (*p* < 0.0001). Patients with M0 and M1 stage disease presented with respective median OS durations of 17 months and 6 months (HR 0.47, 95% CI 0.39–0.56, *p* < 0.0001). Patients who underwent surgical tumor resection had significantly improved survival relative to patients who did not undergo surgery (41 vs. 6 months, HR 0.34, 95% CI 0.29–0.39, *p* < 0.0001), while patients who were treated through chemotherapy experienced significantly improved survival relative to patients who did not undergo chemotherapy (10 vs. 4 months, HR 0.82, 95% CI 0.70–0.96, *p* = 0.0095). Patients with lower grade tumors (I+II) also exhibited significant improvements in OS relative to individuals with higher grade disease (III+IV) (36 vs. 9 months, HR 0.59, 95% CI 0.46–0.77, *p* = 0.0004). The median OS of patients with tumors in the upper, middle, and lower lobes of the lung was 7.5 months, 20.5 months, and 9 months, respectively (*p* < 0.0001). We also found that LSRCC patient survival outcomes were unrelated to patient sex, marital status, or lung laterality (*p* > 0.05).

We then performed univariate and multivariate analyses to identify predictors of patient OS. Univariate analyses suggested that an age ≥80 years was associated with a poor prognosis, whereas factors associated with better patient outcomes included middle lung lobe localization, chemotherapeutic treatment, surgical treatment, Grades I-II, T1, N0-1, and M0 stage disease (Table 2). Age at diagnosis, tumor primary lobe, grade, summary stage, T stage, N stage, M stage, primary site surgery, radiation, and chemotherapy treatment were then included as potential covariates in subsequent multivariate analyses which revealed age at diagnosis, primary site, tumor grade, summary stage, T stage, surgery, and chemotherapy were independently associated with LSRCC patient prognosis (Table 2). This analysis confirmed that patients ≥80 year old had significantly worse outcomes, in line with the results of the above univariate analysis.

**TABLE 2:**
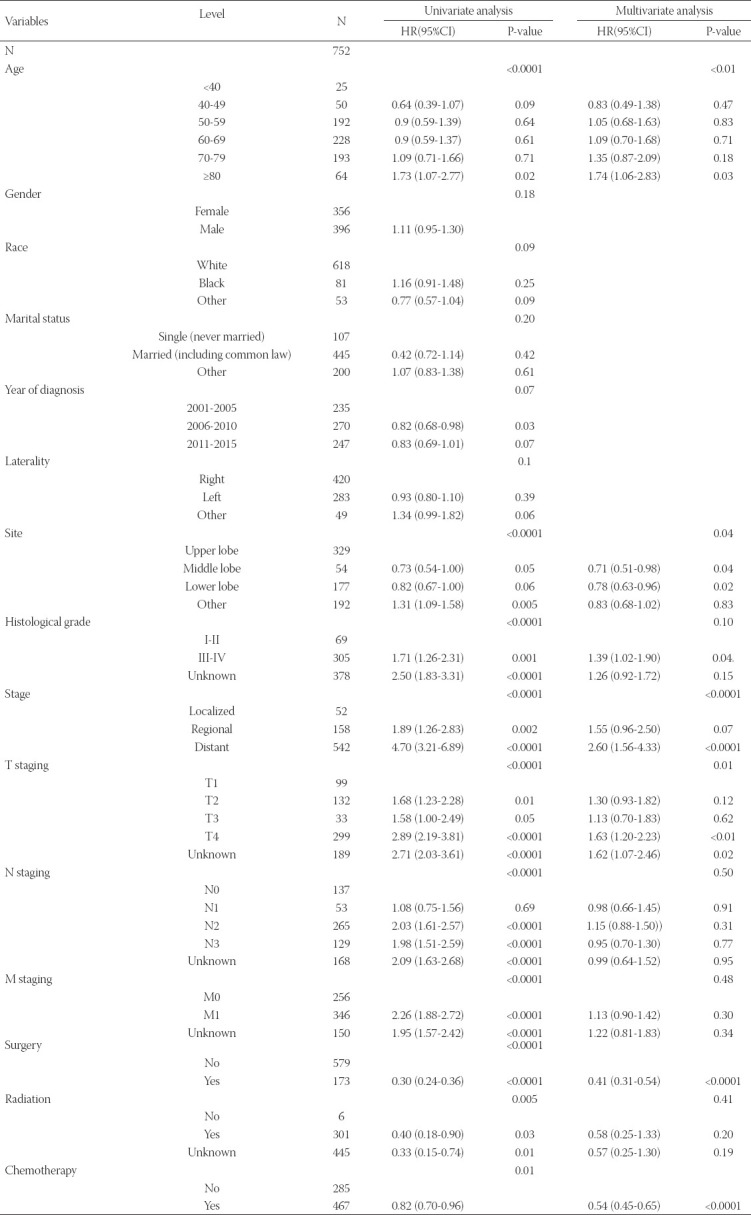
Univariate and multivariate analyses of the median overall survival of LSRCC patients

## DISCUSSION

Mucin-producing adenocarcinomas of the lung can be differentiated into SRCC, solid adenocarcinomas with mucin production (SA), mucinous bronchioloalveolar carcinomas, mucinous (“colloid”) adenocarcinomas, mucinous cystadenocarcinomas, and mucoepidermoid carcinomas [10]. SRCC tumors are unique and highly invasive mucin-producing adenocarcinomas containing high levels of cytoplasmic mucin, resulting in the displacement of the nucleus. Given the rarity of LSRCC, most descriptions of these cases are limited to individual case reports or case series. It is vital that these cases be analyzed in detail to identify the clinicopathological characteristics of this disease and to identify factors associated with patient survival outcomes. As such, we herein utilized the SEER database to comprehensively analyze the clinical features of LSRCC.

LSRCC incidence rates were just 0.14–1.5% among previously reported cases [6,11]. Differences in LSRCC incidence rates among reports may be due to differences in the definition of this disease type, as the diagnostic criteria are not firmly established, and the SRCC component used to define these tumors has varied from 5 to 50% in prior reports [6,12,13]. Consistent with prior findings, we observed no significant differences in race, ethnicity, or marital status when comparing LSRCC and LMAC patient cohorts in the SEER database.

Prior analyses have revealed that LSRCC patients are younger on average than are non-SRCC patients [12,14]. In line with these findings, we observed significantly fewer patients over the age of 70 in the LSRCC patient cohort relative to the LMAC patient cohort (34.2% vs. 47.1%), and we found that LSRCC patients had a lower mean age than did LMAC patients (63.8 vs. 67.5 years). LSRCC patients were also more likely to be male than were LMAC patients (52.7% vs. 42.5%, *p* < 0.05). Tsuta et al. found that most LSRCC tumors were located in the left upper lobes of the lung [12]. We similarly found that lower lobe tumors were rarer in the LSRCC cohort relative to the LMAC cohort (23.5% vs. 39.7%), although there were similar frequencies of tumors of primary origin in the left lung lobe between these two cohorts (37.6% vs. 40.3%).

The vascular invasion, lymphatic invasion, and lymph node metastasis rates in cases where the SRCC component was ≥50% were significantly higher than those in non-SRCC groups [12]. Consistent with these prior findings, we determined that the relative frequencies of patients with distant summary stage, T4 stage, N3 stage, and M1 stage disease in the LSRCC patient cohort were significantly higher than those of patients in the LMAC patient cohort (72.1% vs. 45.8%, 39.8% vs. 23.7%, 17.2% vs. 5.9%, and 46.0% vs. 30.8%, respectively).

Due to the invasive nature of SRCC, patients generally have a poor prognosis attributable to the diffuse invasive nature of the tumors resulting in widespread metastasis at time of initial diagnosis. The clinicopathological characteristics and survival outcomes associated with LSRCC are reported to be unique [14,15]. However, the limited number of published cases to date has made the prognosis of this cancer subtype relatively unclear. In one prior analysis, SRCC+ adenocarcinoma patients experienced significantly poorer outcomes relative to SRCC-adenocarcinoma patients (median survival time 46.7 months vs. 90.0 months, *p* = 0.0319) [16]. We similarly determined that LSRCC patients had a poorer prognosis than LMAC patients (mOS 8 vs. 18 months, HR 1.62, 95% CI 1.47-1.79, *p* < 0.0001). However, after adjusting for potential confounding variables, we detected no significant differences in OS between these two patient cohorts, suggesting that poor differentiation, more advanced tumor stage, and lower rates of chemotherapy and surgery are associated with poorer LSRCC patient prognosis. The lack of significance between groups may be attributable to the small sample sizes of TOS.

Prior reports evaluating non-small-cell lung cancer (NSCLC) patients found that age, gender, smoking status, stage, histology, and surgical treatment were all independent predictors of individual outcomes [17-21]. In our analysis, we determined that age ≥80 years was associated with poorer LSRCC patient survival, whereas middle lobe localization, tumor Grades I-II, T1, having undergone surgery, and having undergone chemotherapy were all significant predictors of better survival outcomes. Our results were thus consistent with those pertaining to NSCLC patients. Tumor stage is an important prognostic factor associated with many tumor types. In addition, our determination that early diagnosis and interventional treatment were associated with better LSRCC patient prognosis is in line with the previous studies [22]. As such, LSRCC can be most effectively treated through surgery and chemotherapy. Unexpectedly, our multivariate analysis suggested that radiotherapy was not associated with any significant improvement in patient survival, although this may be due to the large percentage of patients for whom radiotherapy treatment status was unknown. We found that gender was not significantly associated with LSRCC patient outcomes, potentially due to the higher frequency of EGFR mutations among female patients such that they benefitted more significantly from EGFR inhibitors, resulting in improvements in their PFS and OS as shown in a prior study (overall HR = 0.45; 95% CI: 0.32–0.64, and HR = 0.62; 95% CI: 0.48–0.80, respectively) [23].

There are a number of limitations to this analysis. For one, this was a retrospective study that is susceptible to selection bias. In addition, certain known prognostic parameters were not incorporated into our analyses, including thoracic gross tumor volume, tumor size, maximum positron emission tomography standardized uptake values (SUV_max_), vascular invasion, or perineural invasion [24,25]. Similarly, certain relevant molecular factors such as anaplastic lymphoma kinase rearranged and ROS1 rearranged were not assessed [26-28]. The SEER database also lacks any information pertaining to patient comorbidities, drug usage, surgical treatments, or radiotherapy doses, thus limiting our ability to identify factors associated with patient outcomes. In addition, time- and site-specific variability in the entry of data into the SEER database may contribute to significant heterogeneity within this dataset. Despite these limitations, we believe that our results offer new insights that will guide future studies of this rare cancer.

## CONCLUSION

In summary, we determined that the clinical features of LSRCC are distinct from those of LMAC and that the former is associated with a worse prognosis. Early diagnosis and interventional treatment are associated with improvements in LSRCC patient outcomes. Overall, we assessed the clinical characteristics of LSRCC using the SEER database, enabling us to better understand the diagnosis and prognosis of this rare disease.
